# A dual-genome microarray for the pea aphid, *Acyrthosiphon pisum*, and its obligate bacterial symbiont, *Buchnera aphidicola*

**DOI:** 10.1186/1471-2164-7-50

**Published:** 2006-03-14

**Authors:** Alex CC Wilson, Helen E Dunbar, Gregory K Davis, Wayne B Hunter, David L Stern, Nancy A Moran

**Affiliations:** 1 Department of Ecology and Evolutionary Biology, University of Arizona, Tucson, AZ, 85721, USA; 2 Department of Ecology and Evolutionary Biology, Princeton University, Princeton, NJ, 08544, USA; 3 United States Department of Agriculture, Agricultural Research Service, U.S. Horticultural Research Laboratory, Fort Pierce, FL, 34945, USA

## Abstract

**Background:**

The best studied insect-symbiont system is that of aphids and their primary bacterial endosymbiont *Buchnera aphidicola*. *Buchnera *inhabits specialized host cells called bacteriocytes, provides nutrients to the aphid and has co-speciated with its aphid hosts for the past 150 million years. We have used a single microarray to examine gene expression in the pea aphid, *Acyrthosiphon pisum*, and its resident *Buchnera*. Very little is known of gene expression in aphids, few studies have examined gene expression in *Buchnera*, and no study has examined simultaneously the expression profiles of a host and its symbiont. Expression profiling of aphids, in studies such as this, will be critical for assigning newly discovered *A. pisum *genes to functional roles. In particular, because aphids possess many genes that are absent from *Drosophila *and other holometabolous insect taxa, aphid genome annotation efforts cannot rely entirely on homology to the best-studied insect systems. Development of this dual-genome array represents a first attempt to characterize gene expression in this emerging model system.

**Results:**

We chose to examine heat shock response because it has been well characterized both in *Buchnera *and in other insect species. Our results from the *Buchnera *of *A. pisum *show responses for the same gene set as an earlier study of heat shock response in *Buchnera *for the host aphid *Schizaphis graminum*. Additionally, analyses of aphid transcripts showed the expected response for homologs of known heat shock genes as well as responses for several genes with unknown functional roles.

**Conclusion:**

We examined gene expression under heat shock of an insect and its bacterial symbiont in a single assay using a dual-genome microarray. Further, our results indicate that microarrays are a useful tool for inferring functional roles of genes in *A. pisum *and other insects and suggest that the pea aphid genome may contain many gene paralogs that are differentially regulated.

## Background

Ancient and now highly specialized associations with bacterial symbionts are widespread in invertebrate groups that feed on nutrient-poor diets [1]. Such associations are common in insects, in which the best studied symbiotic system is that of aphids (Insecta: Hemiptera: Aphididae) and their primary symbiont *Buchnera aphidicola *[2]. *Buchnera*, which has not been established in laboratory cultures, inhabits specialized host cells (bacteriocytes), is required for nutrition (host development, growth and reproduction) and has been strictly vertically transmitted from mother to offspring for the last 150 million years [2-5]. In addition to being the best studied insect-symbiont system [6], aphids are of general interest due to their economic and agricultural status as plant pests and plant disease vectors [7] and their complex life cycles with multiple developmental pathways and parthenogenesis [8,9].

The pea aphid *Acyrthosiphon pisum *(Insecta: Hemiptera: Aphididae) will be one of the first insect species outside of the Holometabola to serve as a model system in genomics [10]. Currently, little is known about gene expression in this newly emerging model organism. In addition, it is already clear from the growing collection of aphid ESTs that aphids possess many genes that are absent from *Drosophila *and other holometabolous insect taxa [11-13]. Accurate and successful interpretation of the *A. pisum *genome sequence will depend critically on studies of gene expression under different environmental conditions, in different tissues and morphs, and at different developmental stages [14,15].

The complete genome sequences are now available for the *Buchnera *from three aphid species [5,16,17], including *A. pisum *[5]. Facilitated by these genome sequences and other studies, *Buchnera *has become a model for studying the evolution of symbiotic and pathogenic lifestyles in bacteria [6,18,19].

In the present study we take a metagenomic approach [20] to examining transcriptional responses to heat shock in a host/symbiont system, using pooled RNA samples and a microarray containing gene sets from both organisms. Select results were verified with reverse-transcriptase quantitative PCR (RT-qPCR). To our knowledge, this dual-genome microarray is the first used to examine simultaneously the pooled transcriptome of an animal host and its symbiont; a previous dual-genome array has been used to study Rhizobium-plant interactions [21].

The transcriptional response to heat shock has been well characterized in other *Buchnera *[22] and in other insects [23], enabling us to generate *a priori *predictions and thus to assess the success of our array design. Further, aphids are highly sensitive to heat, with considerable variation among species (e.g., [24-27]). Part of this sensitivity stems from the intolerance of the resident symbionts to high temperature. Aphid lineages can be rendered infertile as a consequence of heat-induced elimination of their resident *Buchnera *[28], and field populations of *A. pisum *exposed to naturally occurring high temperatures undergo a decline in the number of bacteriocytes, reflecting the decline of the *Buchnera *population within heat-stressed aphid hosts [29]. Thus, the overall thermal tolerance of this symbiotic system is dependent on both host and symbiont responses to thermal challenge.

To date, there are no published studies on heat shock responses of any aphid genes. Previously, Wilcox *et al*. [22] studied gene expression under heat shock in *Buchnera *derived from the aphid *Schizaphis graminum *and identified a set of nine genes that were significantly upregulated. Six of the nine genes, *mopA *(= *groEL*), *mopB *(= *groES*), *dnaJ, dnaK*, *grpE*, and *ibpA *are heat shock genes in other bacteria such as the related species *Escherichia coli *and are known to function in the recycling and refolding of degraded proteins. Two additional genes, *fpr *and the pseudogene of *yjeA*, showed elevated transcript levels following heat shock. Both are positioned immediately downstream of *ipbA *and its σ^32 ^heat shock promoter, and their response reflects transcriptional read-through based on reverse transcriptase quantitative PCR assays of different regions of the transcripts [22].

## Results and discussion

### Heat shock response in *Buchnera *of *A. pisum*

**Figure 1:**
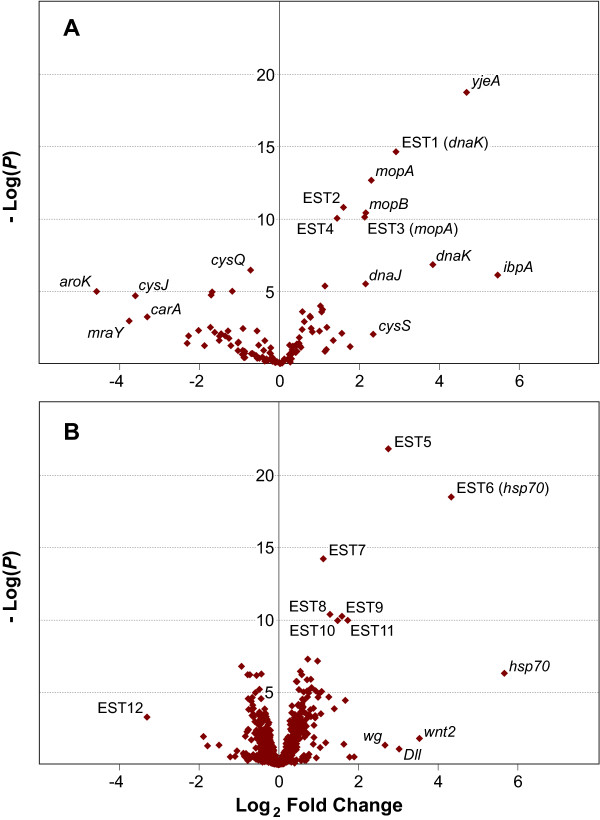
**Fold-change in gene expression after exposure to heat stress**. Line at -Log(*P*) = 5 corresponds to *P *= 0.0001. **A**. *Buchnera *EST1 (*dnaK*) = CN582666, EST2 (*fliI*) = CN587517, EST3 (*mopA*) = CN585306, EST4 (*metE*) = CN582733. **B**. Pea aphid, *A. pisum*. EST5 = CN584139, EST6 (*hsp70*) = CN586725, EST7 = CF588076, EST8 = CN586853, EST9 = CN585958, EST10 = CF587727, EST11 = CN587624, EST12 = CN584928.

Overall, the set of genes responding to heat shock in the *Buchnera *of *A. pisum *is similar to the set previously identified in the *Buchnera *of *S. graminum *[22]. In both, the following genes belonging to the *E. coli *heat shock (HS) regulon were significantly upregulated: *dnaK*, *mopA *(= *groEL*), *mopB *(= *groES*), *ibpA *and *dnaJ *(Figure 1A). (We also detected a similar fold-change in expression of the *E. coli *HS regulon gene, *grpE*, in *Buchnera *of *A. pisum *as reported for *Buchnera *of *S. graminum*, but this change was not statistically significant.) In *Buchnera *of both aphid species, *yjeA *is downstream of *ibpA *and *fpr*, but encoded on the opposite strand. Our results show an increase in *yjeA *transcript with heat shock in *Buchnera *of *A. pisum*, as described for *Buchnera *of *S. graminum*. Because the microarray probes consist of double-stranded amplicons, the most likely interpretation is that the heat-shock induced transcription of *ibpA *continues through both *fpr *and *yjeA*, resulting in elevated transcription of the non-coding strand of *yjeA *(the gene *fpr *is not present on our array). We verified this explanation for *yjeA *transcription under HS through targeted RT-PCR of a region overlapping *ibpA*, *fpr*, and the complement of *yjeA *(data not presented), using the same approach applied to *Buchnera *of *S. graminum *[22].

In addition to the heat-responsive genes identified for *Buchnera *of *S. graminum *[22], two other *Buchnera *probes showed increased signal following HS (Figure 1A): EST2 (*fliI*), flagellum-specific ATP synthase and EST4 (*metE*), 5-methyltetrahydropteroyltriglutamate-homocysteine S-methyltransferase. The gene *metE *was present as two types of probe: one, EST4, showed consistent increase in signal under HS, and the other, a directly amplified product from genomic DNA, did not respond. Our interpretation is that the spot corresponding to EST4 was contaminated with some other sequence, and that *metE *is not responding.

Signal from several *Buchnera *genes was decreased following HS (Figure 1A). These include *aroK*, *mraY*, *cysJ*, *carA *and *cysQ*. Of these, *carA *also showed down-regulation under HS in the study of *Buchnera *of *S. graminum *[22].

**Figure 2:**
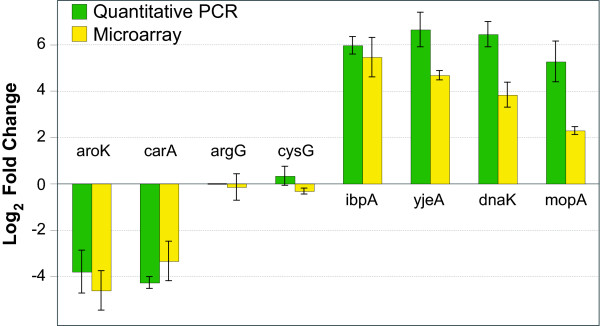
**Mean fold-change estimates for select *Buchnera *of *A. pisum *genes compared between microarray and RT-qPCR methods**. Standard error bars are shown. RT-qPCR data have been standardized relative to *argG*.

Using RT-qPCR, to investigate the accuracy of our microarray results for a select set of eight *Buchnera *genes, we found good congruence between methods, using the non-HS genes *argG *and *cysG *as controls (Figure 2, data for *cysG *not shown).

While the set of genes responding under HS corresponded to the set identified for *Buchnera *of *S. graminum*, the magnitude of response was much larger for *Buchnera *of *A. pisum*, both from the microarray and the RT-qPCR estimates. The HS treatment was the same for both studies; yet, expression changes ranged from under 2-fold to 13-fold in *Buchnera *of *S. graminum *[22] compared with 5-fold to 70-fold in *Buchnera *of *A. pisum *(Figure 1A, Figure 2).

### Heat shock response in *A. pisum*

Two paralogs of the HS gene, *hsp70*, were included on the array, one derived from a PCR product amplified from genomic DNA (*hsp70*) and the other derived from an EST (Figure 1B, EST6 – CN586725). Both showed greater than 16-fold increase in transcript abundance under HS (Figure 1B). Seven other genes (Figure 1B, EST5 and EST7 – EST12) showed large fold-changes and/or statistically significant changes in gene expression under HS conditions.

We investigated the accuracy of our microarray results using RT-qPCR for eight *A. pisum *genes representing a range of responses on the array. Two of these showed no change on the array and were used as control genes (EST13 (CN587115, a homolog of *EF1a*) and EST14 (CN582657)), and both control genes gave similar results. We found good congruence between microarray and RT-qPCR methods for aphid EST 6 (a homolog of *hsp70*) (log_2 _fold-change estimate from microarray data: 5.67 ± S.E. 0.87 *c.f*. log_2 _fold-change estimate from RT-qPCR: 7.32 ± S.E. 1.03). However, RT-qPCR indicated no significant change in relative expression for five of the aphid ESTs that showed either upregulation (EST5, EST7, EST8 and EST10) or down-regulation (EST12) under HS. This indicates that expression of the designated gene (corresponding to the EST and to the sequence matching the PCR primers), does not change under HS. Two explanations are (1) that a paralogous gene is undergoing change in expression giving cross-hybridization on the array but not giving PCR product because of the greater stringency of the RT-qPCR and (2) that there is contamination of the arrayed spots attributable to splash during transfers to the microtiter plates or carry-over of the array pins.

To discriminate between these two possibilities, we performed a dye-flip microarray hybridization experiment using samples differing only in the presence or absence of added amplicons of known HS genes represented on the array. RNA representing the transcript pool of aphids under control conditions was divided into two portions, one spiked with amplicons of four upregulated *Buchnera *genes (*dnaK*, *ibpA*, *mopA*, *yjeA*) and one spiked with the amplicon of an upregulated aphid gene (*hsp70*). Two of the five aphid genes that showed incongruent results (EST8 and EST10) did not change expression in the spike experiment. This indicates that the observed microarray results from the HS experiment cannot be explained as the result of contamination among probes on the array. Instead, they likely reflect cross-hybridization of closely related gene transcripts [30].

Non-target sequences with >70% sequence identity can cross-hybridize to cDNA probes [31]. This is illustrated on our array, which contains three members of the aphid *hsp70 *gene family; *hsp70*, which was amplified using PCR primers from genomic DNA for inclusion on this array and for which we have a cloned PCR product; EST6, which was isolated from EST libraries (Figure 1) and EST15 (CN584562), which shows homology to *hsp70 *and *hsp68 *from other insect taxa and did not show significant upregulation under HS conditions (Table 1). PCR primers designed to EST6 do not amplify our cloned *hsp70 *product or EST15; however, in the spike experiment, *hsp70 *PCR product hybridized to itself and to both the EST6 and EST15 probes (Table 1). Similarly, expression changes in paralogous genes are the most evident explanation for the change in signal of EST8 and EST10 (Table 1). The final two aphid ESTs, EST5 and EST7, did show significant "upregulation" in the spiked pools (Table 1). The EST5 probe cross-hybridized with *hsp70 *and the EST7 probe with one of the four *Buchnera *genes. We could not find any significant stretches of sequence similarity to explain these results, suggesting that these probes were contaminated. The EST5 and EST7 results are most readily attributable to splash on the microtiter plates. However, results from the spike experiment (Table 1) clearly indicate that apparent upregulation of *wg*, *wnt2 *and *Dll *(fold-changes of 6–12 under HS, without strong statistical significance (Figure 1B)) resulted from pin carryover of the *hsp70 *probe during the printing process. The same pin stamped *hsp70*, *Dll*, *wg *and *wnt2*, in that order.

### Usefulness of EST-based microarrays for functional annotation of the aphid genome

These results suggest that, in addition to *hsp70*, paralogs of EST8 and of EST10 undergo significant upregulation under HS. Both have homologs in many other taxa, but we found no reports of involvement in the HS response in other organisms. EST8 shows significant homology to tyrosyl-tRNA synthase from *Homo sapiens*, *Mus musculus *and *Drosophila virilis*, while EST10 is homologous to the gene encoding the molybdenum cofactor of sulfurase in *Bombyx mori*, *Bos taurus *and *Homo sapiens*. Molybdenum cofactor sulfurase is responsible for xanthine dehydrogenase activation in various organisms, a gene which plays a critical role in nitrogen metabolism, especially in insects [32]. Our results suggest that genes that share significant sequence similarity to these two ESTs have a function in the heat stress response in aphids.

## Conclusion

One of the outstanding questions in host/symbiont systems is how gene expression in the partner organisms is coordinated. EST studies of aphid bacteriocytes have shown elevated expression of genes associated with a range of cellular functions including amino acid metabolism, cell transport, and antibacterial activity [13]. The presence of these functional categories at high frequency in bacteriocyte EST libraries highlights important aspects of genome coordination between symbiont and host. Further progress in understanding how and when these genes are upregulated and the roles they play in mediating the symbiosis will require extensive expression studies using arrays such as the one we describe here.

We have demonstrated the feasibility of using a dual-genome microarray to simultaneously study the expression profile of an aphid and its obligate bacterial symbiont. The success of this approach is dependent on the relative abundances of transcripts from the partner organisms. *Buchnera *occurs in large numbers within aphids; their genomes constitute about 5–10% of total DNA content [3]. A quantitative PCR study of gene copy number in intact adult pea aphids indicated a ratio of approximately 7.5 single copy *Buchnera *genes for each single copy aphid gene (H. Dunbar and N. Moran, unpublished data). This estimate is consistent with previous estimates of genome sizes of the two organisms and of the proportion of total DNA belonging to *Buchnera *[3]. Thus, we expected transcript abundances of *Buchnera *and aphid genes to be comparable, enabling hybridization of the pooled sample on the same array without loss of signal for either organism. Use of a dual-genome array for the study of symbiosis in systems where symbionts are found in low copy number, *e.g. Wolbachia*, the facultative bacterial endosymbiont of *Drosophila *and other insects [33], is likely to prove more difficult. However, many animals, including many other insects and marine invertebrates, do possess large numbers of obligate primary symbionts. In these composite organisms, an understanding of the integration of component genomes requires the synchronous examination of gene expression in each member.

Finally, our results for the pea aphid genes suggest that the pea aphid genome may contain many gene paralogs that are differentially regulated. Preliminary analysis from an EST project suggests that the pea aphid genome may contain many gene duplications [34]. This fact will complicate analysis of differential gene expression in pea aphids using cDNA microarrays, and suggests that establishing the differential regulation of specific genes will require qPCR. The full extent of gene paralogy in the pea aphid will soon be revealed by whole genome sequencing [10].

## Methods

### Microarray design

We designed a 7,448 spot, dual-genome microarray containing 1,862 genes. The array includes 76 *Buchnera *genes representing the *E. coli *heat shock (HS) regulon (*clpX, dnaJ, dnaK, grpE, hflB, hslU, hslV, htpX, htrA, ibpA, mopA *and *mopB*), essential amino acid biosynthesis genes (*argA, argB, argC, argD, argE, argF, argG, argH, aroA, aroB, aroC, aroD, aroE, aroH, aroK, carA, carB, cysD, cysG, cysH, cysI, cysJ, cysK, cysN, cysQ, dapA, dapB, dapD, dapE, dapF, glyA, hisB, hisC, hisD, hisF, hisI, ilvC, ilvH, ilvI, leuA, leuD, lysA, metE, pheA, thrA, thrB, trpA, trpC *and *trpG*) and a number of other genes representative of various functional categories such as cold shock, cell wall biosynthesis and DNA repair (*cspC, cysS, endA, lgt, mraY, mrcB, mrsA, murC, murE, murF, nlpD, prfB, serC, ung *and *yjeA*). The array also includes, nine aphid developmental and heat shock genes (*distal-less*, *eyeless*, *fused*, *nanos*, *vasa*, *vasa-like*, *wingless*, *wnt2 *and *hsp70*) and 1777 *A. pisum *ESTs (GEO Accession: GSE3742). All *Buchnera *genes were amplified using gene-specific primers designed from the *Buchnera *of *A. pisum *genomic sequence (GenBank Accession: BA000003, [6]). Primers were designed, where gene length permitted, to give an optimal product of 1000 base pairs with a melting temperature of 55°C. If gene coding sequences were less than 1000 base pairs, primers were designed to maximize product length. We amplified *Buchnera *genes in two 50 μl PCR reactions from a laboratory line of *A. pisum*. This line, 7-2-1 was collected in August 2001 in Cayuga County, New York from alfalfa by Jacob Russell. Since its collection, 7-2-1 has been maintained in continuous parthenogenetic culture in the laboratory of NAM.

All *A. pisum *ESTs were derived from a cDNA library constructed in the laboratory of DLS (described in [34], GenBank Accessions: CF587442 - CF588411 and CN582088 - CN587684). ESTs were clustered in a unigene set and 1824 genes were selected for inclusion based primarily on significant tblastx scores to other eukaryotes when used as queries in searches of the GenBank nr database. The corresponding cDNAs were amplified using T3 and T7 primers to pBluescript.

Genes were arranged in duplicate spots on slides, with *Buchnera *genes grouped into the first and second of 24 subarrays. In addition to spot duplication, the entire set was printed twice on each slide thus; each spot was printed in quadruplicate.

### Heat shock

A single isofemale line of *A. pisum *was used in all HS experiments. This clone, called "TUC", was collected by NAM from fava bean in a garden in Tucson, Arizona in June 1999 and has been maintained in continuous parthenogenetic culture on fava bean at 20°C in the laboratory of NAM since its collection. Clone TUC, in addition to harboring the primary aphid symbiont, *Buchnera*, is host to the secondary bacterial symbiont, *Candidatus *Serratia symbiotica, previously known as the "R-type" [35].

Six to ten adult *A. pisum *were placed on a single fava bean plant in a cup cage and allowed to reproduce at 20°C 16 L:8 D. After 24 – 48 hours the adult aphids were removed from plants and flash-frozen in liquid nitrogen. First generation aphids were then maintained for one week, the developmental period required to attain the adult stage. Heat shock experiments were carried out 1 – 2 days following ecdysis to adulthood. Prior to HS, 10 – 20 control aphids were removed from each plant, flash-frozen in liquid nitrogen and stored at -80°C until RNA extraction and microarray hybridization. Heat shock conditions were applied by ramping from 20°C to 36°C over a two hour period at a rate of 2°C/15 min and then maintaining aphids at 36°C for an additional 2 hours (4 hours in total). At 4 hours cultures were removed from the growth chamber and aphids were quickly collected into Eppendorf tubes, flash-frozen in liquid nitrogen and stored at -80°C until processing. Two biological replicates of clone TUC were exposed to heat shock in parallel.

### RNA extraction and microarray hybridization

We extracted total RNA from control and HS samples using an RNeasy Mini Kit (Qiagen Cat. No.74104). RNA quantity and quality was assessed using an RNA LabChip^® ^on the Agilent 2100 Bioanalyzer. We constructed cDNA from 10 μg of total RNA using Omniscript Reverse Transcriptase and reaction buffer (Qiagen), 6 ng random hexamer primers, 0.5 mM each dATP, dCTP, dGTP, 0.3 mM dTTP, and 0.2 mM amino-allyl linked dUTP. RNA was hydrolyzed, and purified cDNA populations were coupled to Cy3 or Cy5 fluors (Amersham). We flipped dye labels between treatments across experimental replicates. Therefore, results from 4 total slides were analyzed. Purified, labeled cDNA populations were suspended in 80 μl Sigma Hybridization Buffer with 20 μg Human COT1 DNA. Microarray slides were rehydrated, UV crosslinked, and denatured immediately before hybridization. Target samples were combined, denatured for 10 min at 94°C, and pipetted into sealed, microvolume slide manifolds. We ran hybridizations in a GeneTAC automated station at 47°C for 16 hours and programmed 3 washes of variable-stringency SDS/SSC solutions (Sigma).

### Analysis

Signal intensities were measured using spot-finding software, softWoRx Tracker (version 2.20, MolecularWare Inc). Raw median spot intensity and median background intensity data were exported; median background signal intensities were subtracted from median signal intensities. A zero signal value was substituted for any negative signal intensities and all non-zero values were log_2_-transformed. In order to compensate for differences in expression levels between the two genomes represented on the array we analyzed each species separately, by normalizing across probes for each genome. For each species we imported log_2_-transformed data into SAS version 9 and used 'PROC MIXED' to model and remove systematic sources of variation contributed by Cy3 versus Cy5 dyes, slide and hybridization effects, slide × dye interactions and slide × treatment interactions [36,37]. Statistical residuals generated from the normalization step were subjected to mixed-model analyses of variance (log_2 _normalized data for this experiment can be accessed at GEO Accession: GSE3742). We regarded as potentially meaningful any response that showed fold-change greater than four or that had *P*-values less than 0.0001.

### Verification of expression with RT-qPCR

We verified microarray results with real-time quantitative PCR from cDNA from both biological replicates used for microarray hybridization for four of the *Buchnera *genes that appeared to be upregulated under HS conditions (*dnaK*, *ibpA*, *mopA*, and *yjeA*), two *Buchnera *genes that did not show a HS response (*argG *and *cysG*) and two *Buchnera *genes that appeared to be down-regulated under HS conditions (*aroK *and *carA*) as well as five aphid ESTs that appeared to be upregulated under HS conditions (EST5, EST6: *hsp70*, EST7, EST8 and EST10, Figure 1b), two aphid genes that did not show a heat shock response (EST13: *ef1α *and EST14), and one aphid EST that appeared to be down-regulated under HS conditions (EST12, Figure 1b) following the protocol described in [38]. Primers were designed to amplify short regions of these genes and were as follows:

*argG*: F 5' TTCACTATGTCATGGTGCTACTG 3' and R 5' TGCCAGGAATTTTCATCTTTAC 3'; *aroK*: F 5' CTGGGAAAAGCACTATTGGTC 3' and R 5' ACCTCCTCCTGTAGCAAGAAC 3'; *carA*: F 5' TTGCGATTACGCTATTCATGC 3' and R 5' CCTTAACAGGATGGTTGCCTC 3'; *cysG*: F 5' AGGTGGTGATCCCTTTATTTTC 3' and R 5' GAGTATTTGCGATGTGTCAGTG 3'; *dnaK*: F 5' ATGGGTAAAATTATTGGTATTG 3' and R 5' ATAGCTTGACGTTTAGCAGG 3'; *ibpA*: F 5' CCAATATCTGATACACCGAC 3' and R 5' TGAACAGATATATCTAATTCTTTTTC 3'; *mopA*: F 5' ATGTAAAAGACGGAAAAGG 3' and R 5' CCTGCTGGAGAAGAACTAG 3'; *yjeA*: F 5' CGAACAAAAAGGTTGATACCATTC 3' and R 5' TTATTAGCATCAGAAAGCGGATC 3'; EST13 – *ef1α*: F 5' CTGATTGTGCCGTGCTTATTG 3' and R 5' TATGGTGGTTCAGTAGAGTCC 3'; EST6 – *hsp70*: F 5' TAAGAGGAAAACTAAAAAGGACG 3' and R 5' GGAAACTCGGGTGTAGAAATC 3'; EST14: F 5' TATGTTGCAGCAGCCGATAC 3' and R 5' AATGCTGGTCCTTCAGATGC 3'; EST5: F 5' CAAGAAATTCTCCCCTCATTTG 3' and R 5' TGCTCTTGCTAACCCACCAC 3'; EST7: F 5' TTCAAATCGTCGAAGTGTGC 3' and R 5' AAGTCGTCGACCGTGATTTC 3'; EST8: F 5' GCTGTTCTGCGTTCACTTGTAC 3' and R 5' AGTCCGCTATTTTGGATATTGG 3'; EST10 F 5' TTTGGAAGGAATGGACTGTGG 3' and R 5' TTGAGCGTTGATTGTGTGGAC 3'; EST12: F 5' GAAAGAAGATTTGGTGCTTGG 3' and R 5' CTATTATGGGCACTTGCGATC 3'.

### Verification of spot identity with select resequencing across the array

Spots that showed a > 4 fold-change (log_2 _fold-change = ± 2) and/or a significant fold-change (≥ -Log(*P*) = 5), together with the set of ESTs that we annotated through blast-searching as HS genes were resequenced using vector primer, T3, to confirm spot identity. The identity of the majority of resequenced spots (24/36 spots) was verified by a blastn search restricted to the NCBI "EST_other" database [39], which includes the EST sequences for the fragments on our array. A subset of resequenced spots (11/36 spots) returned poor sequence, as expected if more than one template was present. However, three of these, CN585306 (EST3, *Buchnera mopA*), CN586357 (aphid *dnaJ*) and CN586725 (EST6, aphid *hsp70*), were annotated as HS genes and showed large and significant upregulation under HS conditions. The correspondence between gene annotation and change in expression for these genes indicates that the expected gene is present on the array in the correct position. This observation indicates that most elements on the array contain the correct sequence.

### Investigation of cross-hybridization of heat-shock gene products

We investigated cross-hybridization of the most upregulated heat shock genes on our array by spiking control RNA with labeled PCR products corresponding to HS genes. Four *Buchnera *genes (*dnaK*, *ibpA*, *mopA *and *yjeA*) and one aphid gene (*hsp70*) were amplified. PCR reactions were carried out for each gene in two 25 μl volumes containing 50 mM KCl, 10 mM Tris-HCl, 1.5 mM Mg^2+^, 2.5 mM of each dATP, dCTP and dGTP, 1 mM dTTP, 1.5 mM amino-allyl linked dUTP (Sigma A0410-1MG), 0.25 units of Eppendorf Taq DNA polymerase and 10 ng of DNA (*Buchnera *genes) or cDNA (EST6 – *hsp70*). Primers used to amplify genes selected for spiking were as follows: *Buchnera *of *A. pisum dnaK*: F 5' CACCAGAGAGAACTCCTCCC 3' and R 5' ATGGATGGCAATAAACCACG 3', *Buchnera *of *A. pisum ibpA*: F 5' TCAATTAACACTAAGTATTCCTGG 3' and R 5' TAGGTTTTTCCTCTTCCGG 3', *Buchnera *of *A. pisum mopA*: F 5' ACCACAACAGCAACATTATTAGC 3' and R 5' ACCTCCAGCAACTACACCTTC 3', *Buchnera *of *A. pisum yjeA*: F 5' GAAGCGTTTATTAGCATCAG 3' and R 5' TGAACAAGGAGGTAAGCC 3', *A. pisum hsp70*: F 5' GTGTTGATATTTGACCTGGGC 3' and R 5' AATGGTAGAGTTGCGTTCGAC 3'.

We performed a standard dye-flip hybridization experiment using RNA isolated from a control line and spiked with either 20 ng of the aphid *hsp70 *PCR product or 20 ng of each of the four *Buchnera *gene PCR products. Spikes were added to the RNA samples following generation of cDNA and prior to cy3 and cy5 labelling. Thus, for one hybridization, the aphid spike was cy3 labeled and the *Buchnera *spikes were cy5 labeled and for the second hybridization the dyes were reversed. In this way we were able to distinguish between spots that were cross-hybridizing with aphid *hsp70 *versus the *Buchnera *genes. Data from the spike array hybridization experiment were analyzed using the analysis pipeline described above for the heat shock experiments.

## List of abbreviations

EST: expressed sequence tag

HS: heat shock

qPCR: quantitative-PCR

RT-qPCR: reverse-transcriptase quantitative-PCR

## Authors' contributions

DLS provided cDNA libraries. WBH sequenced and provided clones of *A. pisum *ESTs. DLS and NAM designed the array. GKD PCR-amplified aphid ESTs. HED designed primers for and PCR-amplified *Buchnera *of *A. pisum *genes. ACCW and HED executed heat shock experiments and prepared RNA for array hybridization. ACCW annotated the array and analyzed the microarray data. HED designed, executed and analyzed RT-qPCR reactions. ACCW verified spot identity by resequencing. HED, ACCW and NAM designed and executed the spike experiment. ACCW and NAM drafted the manuscript. All authors read and approved the final manuscript.

## Figures and Tables

**Table 1:** Spike microarray hybridization data listing genes showing significant signal increase. Genes that were used to spike the control RNA are marked * for *Buchnera *genes and ** for the aphid gene. Positive log_2 _fold-changes indicate genes that cross-hybridized with aphid *hsp70 *and negative log_2 _fold-changes indicate genes that cross-hybridized with the *Buchnera *genes. Genes reported are those that had log_2 _fold-changes ≥ |1| and a -log(*P*) ≥ 2. Genes are listed in order of signal strength.

Gene	Log_2 _fold-change	Standard Error	-Log(*P*)
hsp70**	7.66	0.29	11.90
EST6 – *hsp70*	4.26	0.29	8.82
*Dll*	2.55	0.37	4.98
*wg*	2.06	0.44	3.36
EST 15 – *hsp70*/*hsp68*	1.84	0.33	4.00
EST5	1.28	0.21	4.35
*wnt2*	1.23	0.34	2.46
CN585728	-1.14	0.16	5.04
EST7	-1.51	0.33	3.28
EST3 – *mopA*	-1.57	0.34	3.29
CN582302	-1.71	0.30	4.13
mopA*	-2.29	0.45	3.79
EST1 – *dnaK*	-4.04	0.39	6.99
dnaK*	-5.48	0.43	7.97
ibpA*	-5.95	0.65	6.30
yjeA*	-7.13	0.17	14.58
